# THE ROLE OF HOSPICE CARE COORDINATION IN THE EXPERIENCE OF LIVE DISCHARGE TRANSITIONS

**DOI:** 10.1093/geroni/igae098.1796

**Published:** 2024-12-31

**Authors:** Cara Wallace, Stephanie Wladkowski, Ruaa Al Juboori, Todd Becker, Joseph Trinitas, Leslie Hinyard, Verna Hendricks-Ferguson

**Affiliations:** Saint Louis University, St. Louis, Missouri, United States; Bowling Green State University, College of Health & Human Services, Bowling Green, Ohio, United States; University of Mississippi, Oxford, Mississippi, United States; Washington University School of Medicine in St. Louis, St. Louis, Missouri, United States; Saint Louis University, St. Louis, Missouri, United States; Saint Louis University, St. Louis, Missouri, United States; Saint Louis University, St. Louis, Missouri, United States

## Abstract

Hospice care improves end-of-life outcomes for patients, while a live discharge results in lost access to supportive services and resources. The quality of this transition is critical for ensuring that a caregiver and patient are prepared for the termination of hospice services, and to avoid negative outcomes, such as a hospitalization or a burdensome transition. This study interviewed 32 caregivers of patients across diagnoses who experienced a discharge from hospice due to decertification—when a patient is removed from hospice due to a stabilized condition. Participants were placed in a priori groups (16 in each group) depending on whether their care transition was more positive (designated by a score of 66.67 or higher on the CTM-15) or more negative (scores of 66.66 or below). There were no statistically significant differences between groups based on demographics. However, qualitative codes relating to care coordination differed based on these a priori groupings. Specifically, the positive transition group was more likely than the negative transition group to report hospice action related to the discharge in the following areas: awareness and information, communication between providers, medication, and receiving support from hospice after the discharge. Alternatively, those in the negative transition group were more likely to report hospice inaction related to awareness and information, communication about the discharge, communication between providers, and lack of help generally. Differences in access to healthcare service and availability of social support and how this impacted quality of life also differed across groups. Research, clinical, and policy implications will be discussed.

